# Supplementary material for: Mosaic cis-regulatory evolution drives transcriptional partitioning of HERVH endogenous retrovirus in the human embryo
Source: eLife. 2022 Feb 18;11:e76257. doi: 10.7554/eLife.76257 (PMC8912925; doi:10.7554/eLife.76257)
Supplement: Supplementary file 4. [file elife-76257-supp4.pdf]

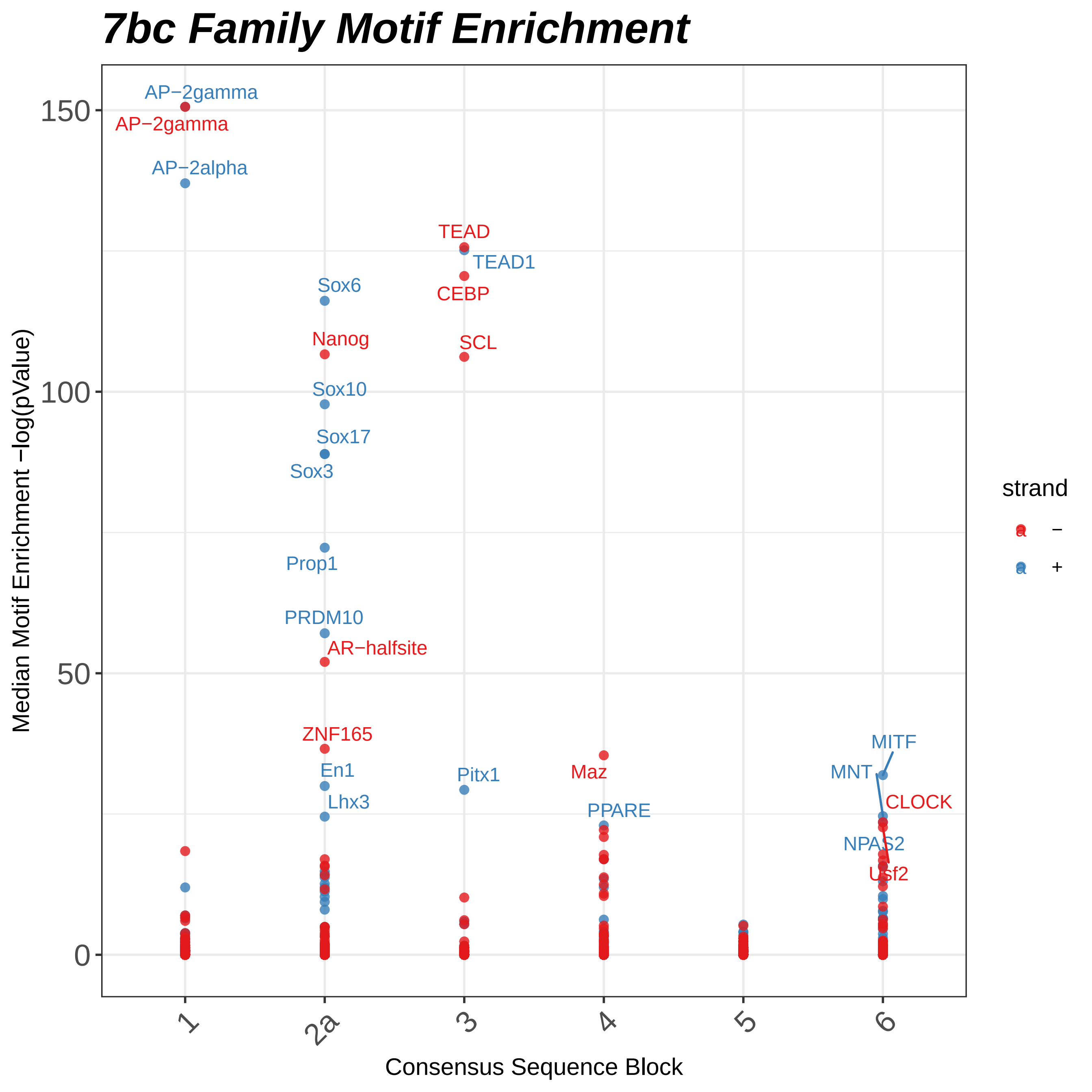
7bc Family Motif Enrichment
Median Motif Enrichment −log(pValue)
Consensus Sequence Block
150
100
50
0
1
2a
3
4
5
6
strand
−
+
AP−2gamma
AP−2gamma
AP−2alpha
TEAD
TEAD1
CEBP
SCL
Sox6
Nanog
Sox10
Sox17
Sox3
Prop1
PRDM10
AR−halfsite
ZNF165
En1
Lhx3
Pitx1
Maz
PPARE
MITF
MNT
CLOCK
NPAS2
Usf2

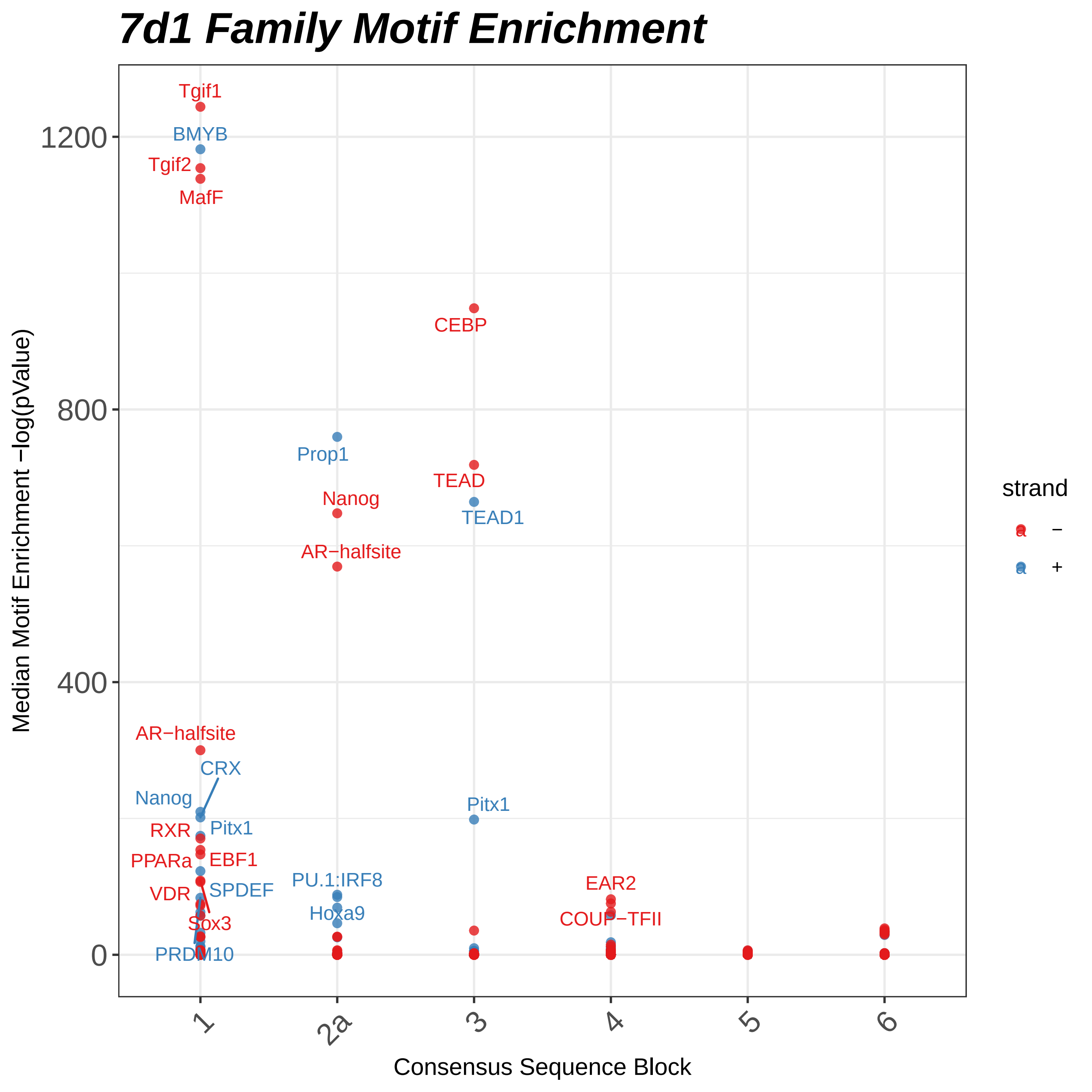

7d1 Family Motif Enrichment
Median Motif Enrichment −log(pValue)
Consensus Sequence Block
1200
800
400
0
1
2a
3
4
5
6
strand
−
+
Tgif1
BMYB
Tgif2
MafF
CEBP
Prop1
TEAD
Nanog
TEAD1
AR−halfsite
AR−halfsite
CRX
Nanog
Pitx1
RXR
Pitx1
PPARa
EBF1
VDR
SPDEF
PU.1:IRF8
EAR2
Hoxa9
Sox3
COUP−TFII
PRDM10

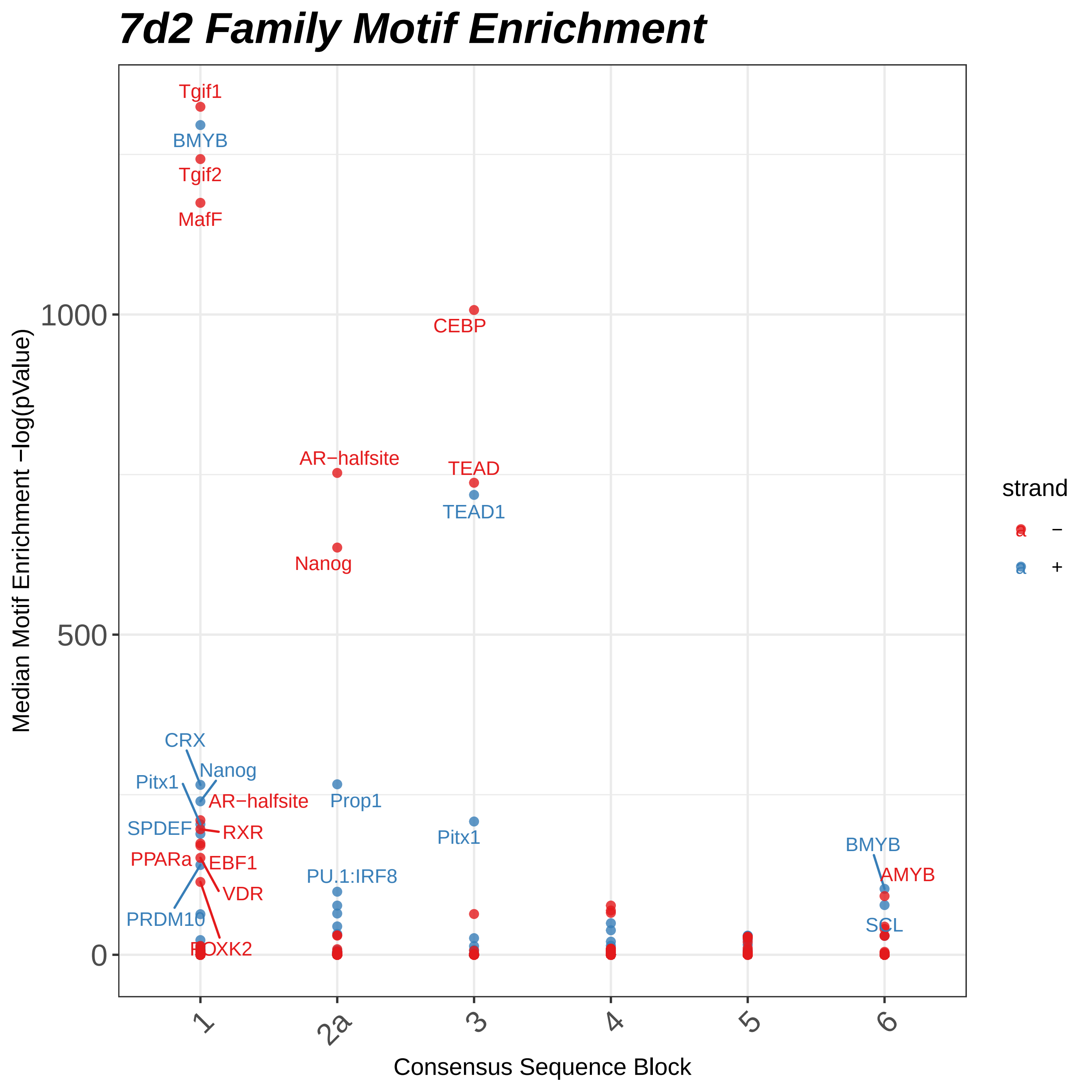
7d2 Family Motif Enrichment
Median Motif Enrichment −log(pValue)
Consensus Sequence Block
1000
500
0
1
2a
3
4
5
6
strand
−
+
Tgif1
BMYB
Tgif2
MafF
CEBP
AR−halfsite
TEAD
TEAD1
Nanog
CRX
Nanog
Pitx1
AR−halfsite
SPDEF
RXR
PPARa
EBF1
VDR
PRDM10
FOXK2
Prop1
Pitx1
PU.1:IRF8
BMYB
AMYB
SCL

7o Family Motif Enrichment

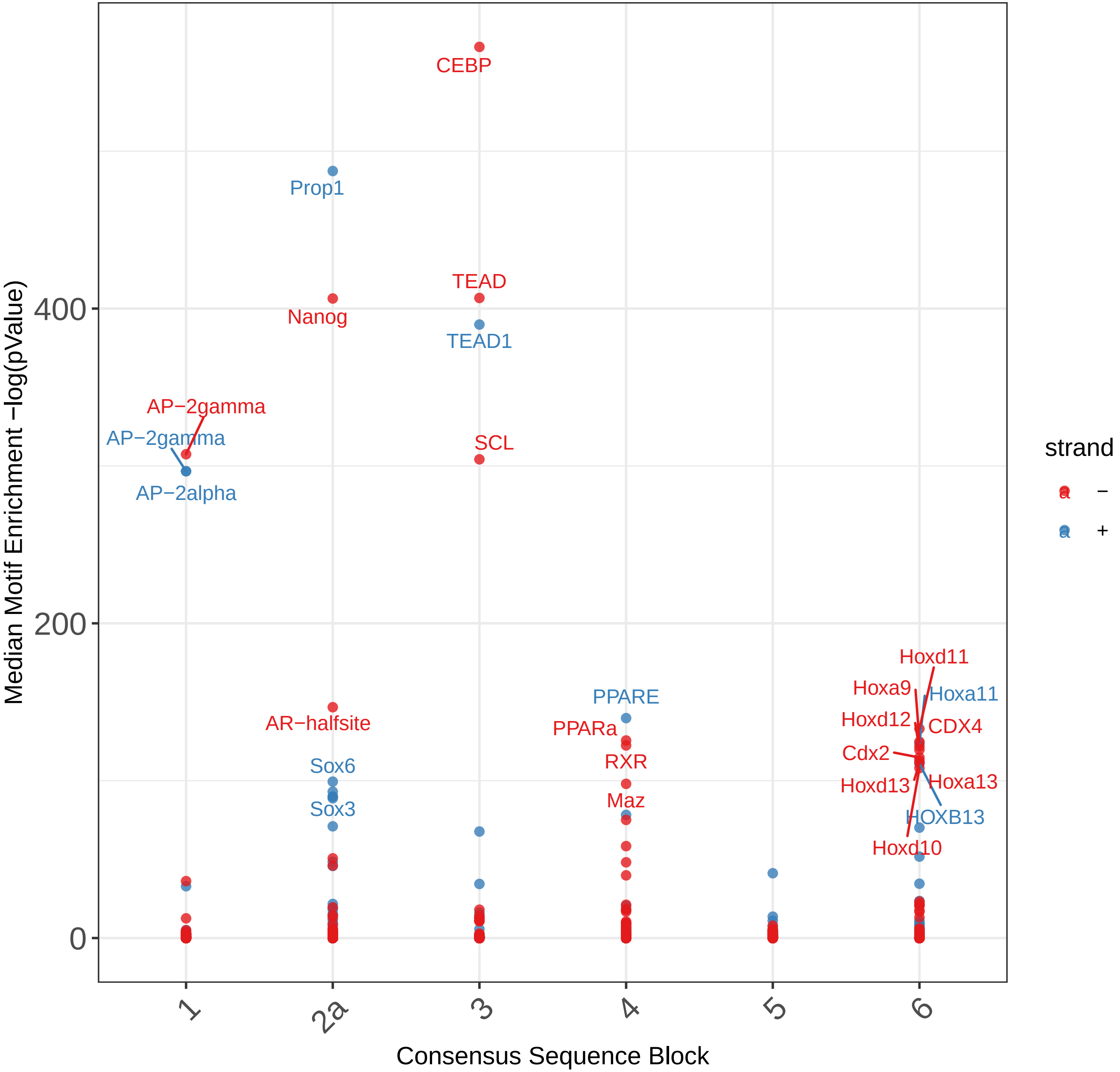

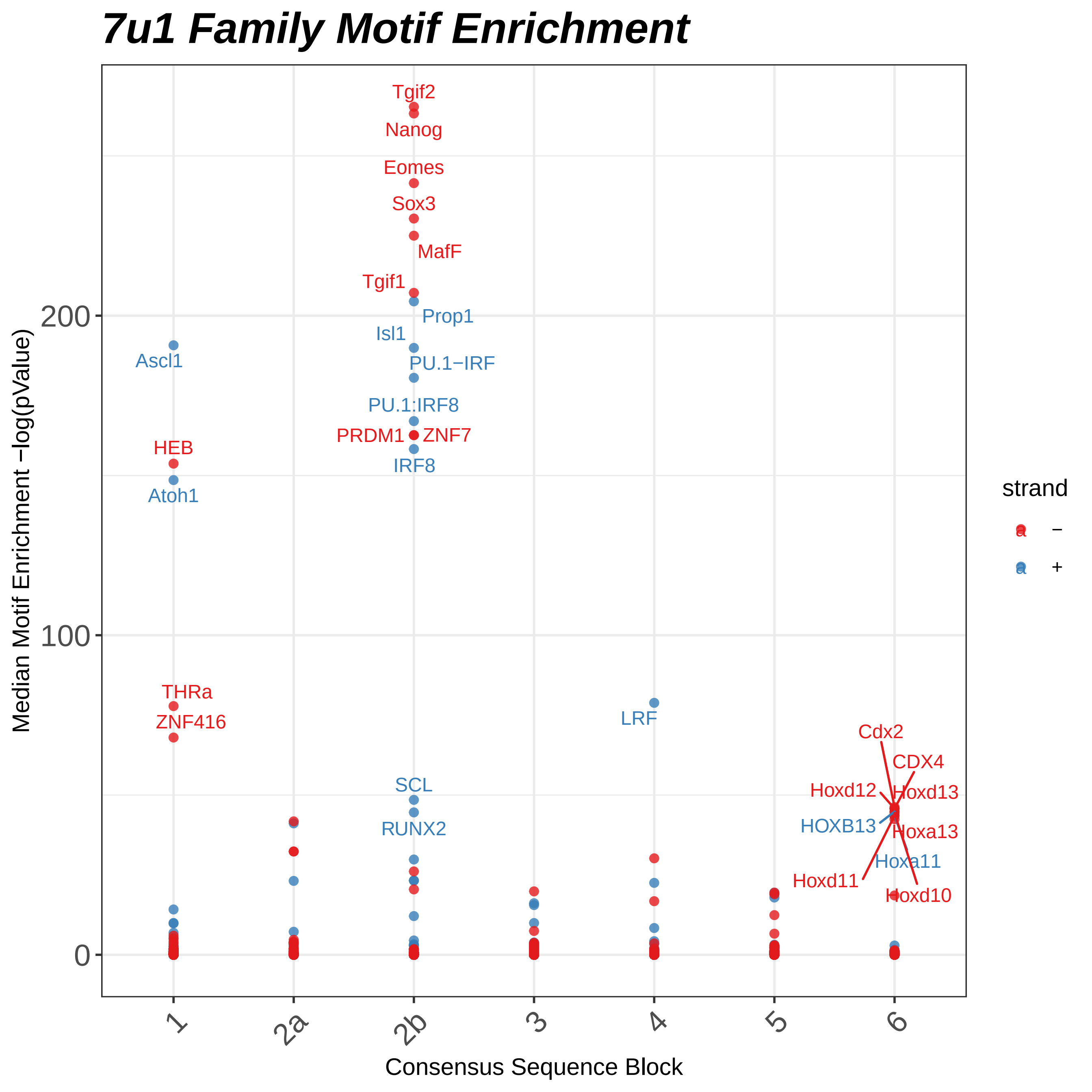

7u1 Family Motif Enrichment
Median Motif Enrichment −log(pValue)
0
100
200
Consensus Sequence Block
1
2a
2b
3
4
5
6
strand
−
+
Tgif2
Nanog
Eomes
Sox3
MafF
Tgif1
Prop1
Isl1
PU.1−IRF
PU.1:IRF8
PRDM1
ZNF7
IRF8
Ascl1
HEB
Atoh1
THRa
ZNF416
LRF
SCL
RUNX2
Cdx2
CDX4
Hoxd12
Hoxd13
HOXB13
Hoxa13
Hoxa11
Hoxd11
Hoxd10

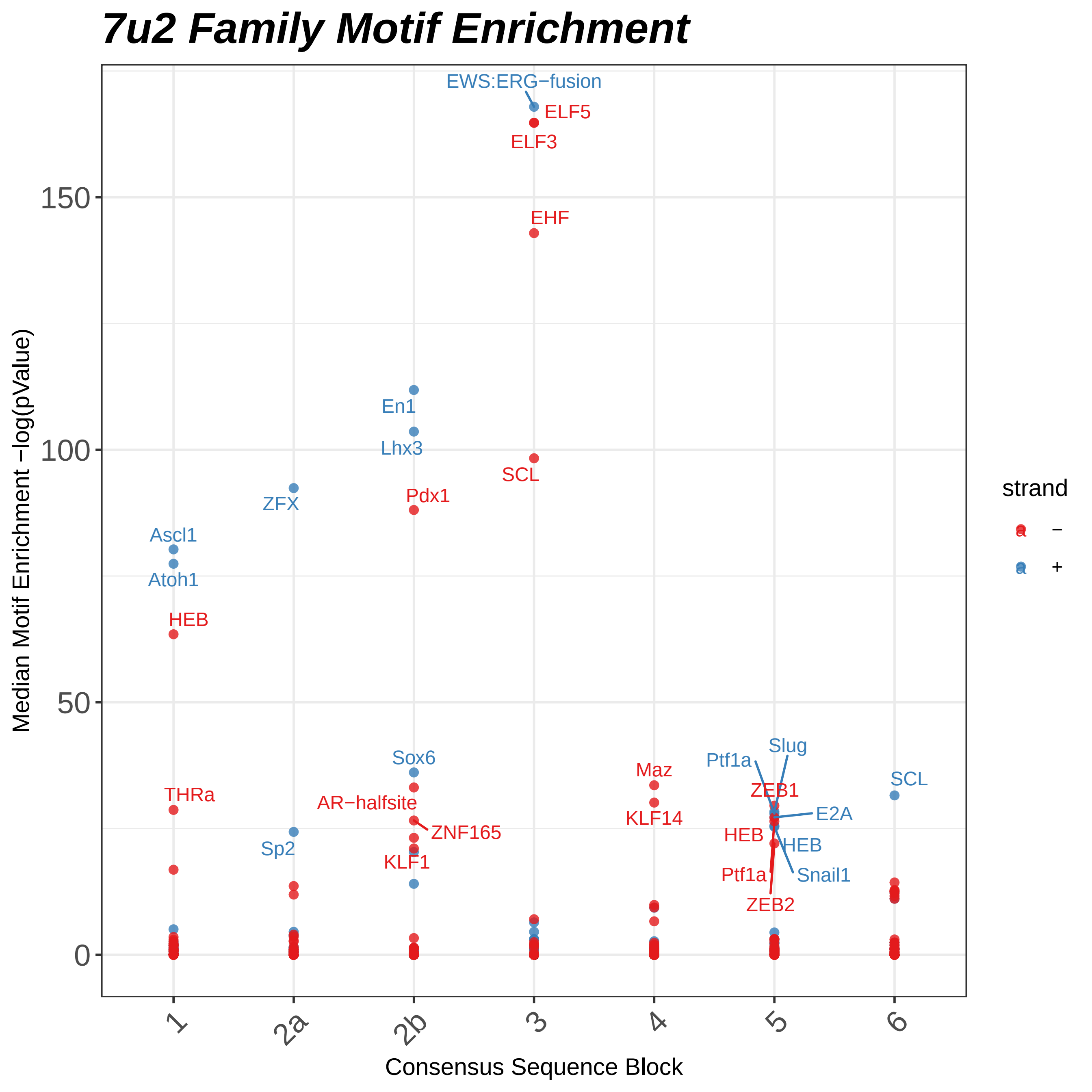
7u2 Family Motif Enrichment
Median Motif Enrichment −log(pValue)
Consensus Sequence Block
strand
−
+
0
50
100
150
1
2a
2b
3
4
5
6
EWS:ERG−fusion
ELF5
ELF3
EHF
SCL
En1
Lhx3
Pdx1
ZFX
Ascl1
Atoh1
HEB
THRa
Sp2
Sox6
AR−halfsite
ZNF165
KLF1
Maz
KLF14
Ptf1a
Slug
ZEB1
E2A
HEB
HEB
Snail1
Ptf1a
ZEB2
SCL

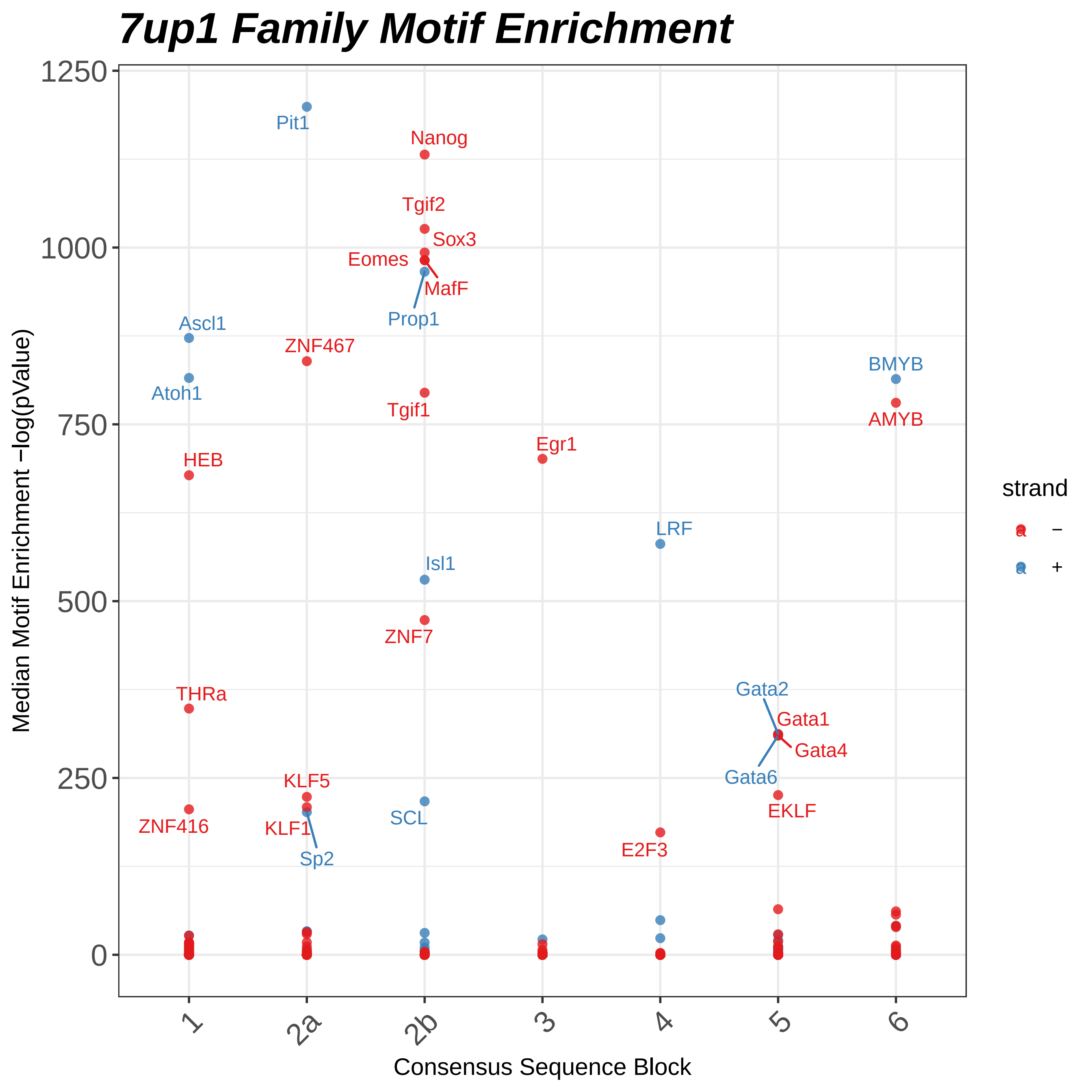

7up1 Family Motif Enrichment
Median Motif Enrichment −log(pValue)
1250
1000
750
500
250
0
Pit1
Nanog
Tgif2
Sox3
Eomes
MafF
Prop1
Ascl1
ZNF467
Atoh1
Tgif1
BMYB
AMYB
Egr1
HEB
LRF
Isl1
ZNF7
THRa
Gata2
Gata1
Gata4
Gata6
KLF5
EKLF
ZNF416
KLF1
SCL
Sp2
E2F3
1
2a
2b
3
4
5
6
Consensus Sequence Block
strand
−
+

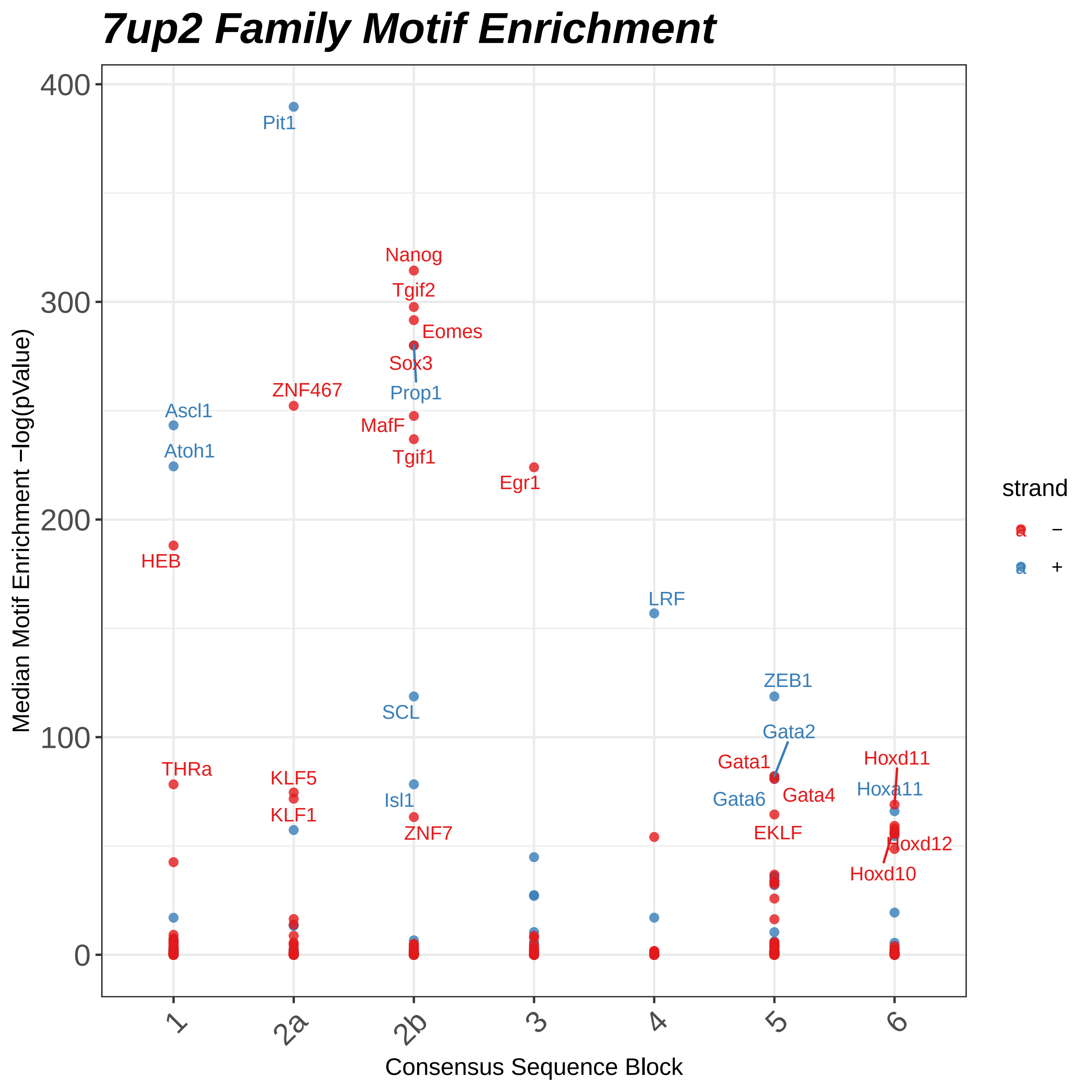
7up2 Family Motif Enrichment
Median Motif Enrichment −log(pValue)
400
300
200
100
0
Pit1
Nanog
Tgif2
Eomes
Sox3
Prop1
ZNF467
Ascl1
MafF
Atoh1
Tgif1
Egr1
HEB
LRF
ZEB1
SCL
Gata2
Gata1
Hoxd11
THRa
KLF5
Hoxa11
Gata4
Isl1
Gata6
KLF1
ZNF7
EKLF
Hoxd12
Hoxd10
1
2a
2b
3
4
5
6
Consensus Sequence Block
strand
−
+

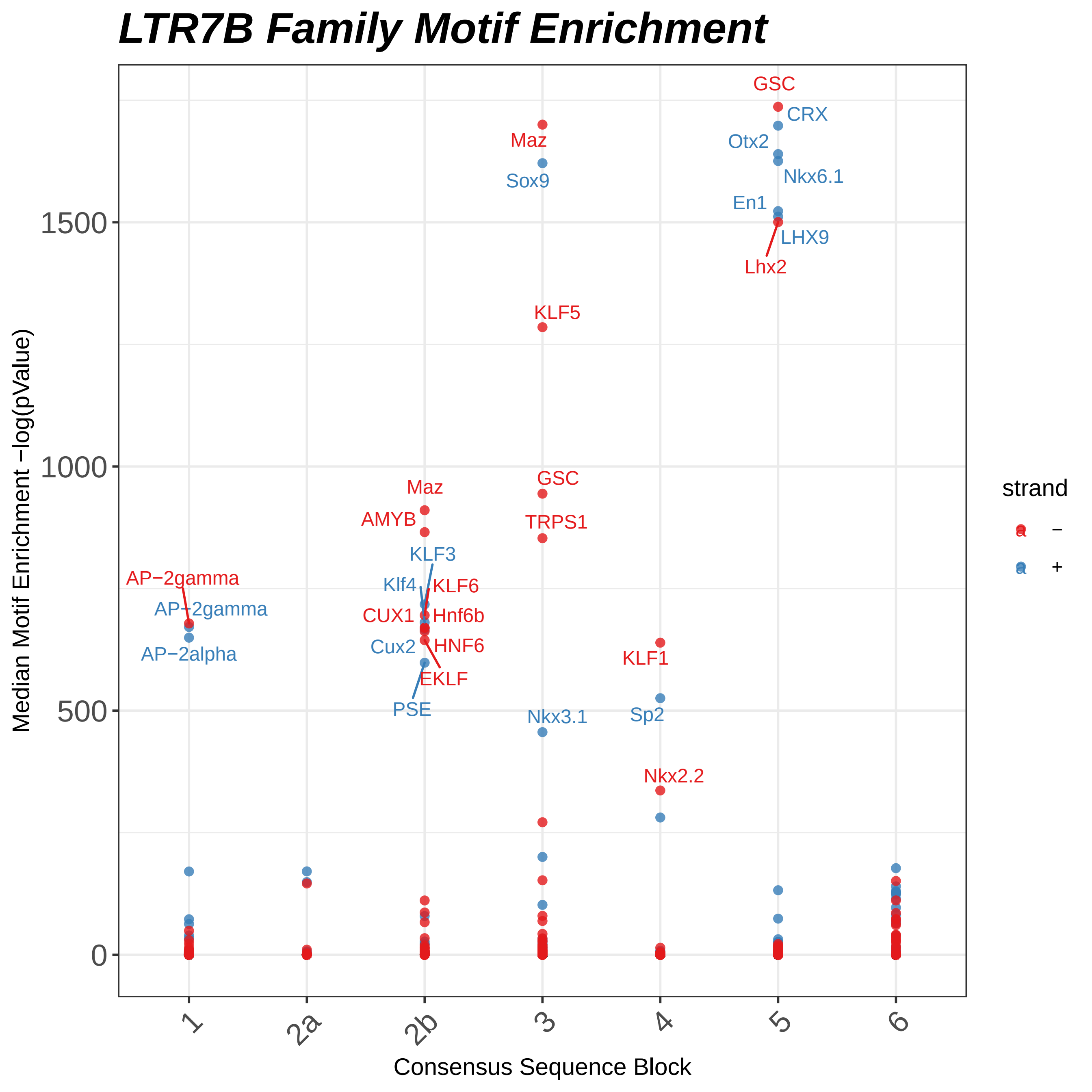
LTR7B Family Motif Enrichment
GSC
CRX
Maz
Otx2
Sox9
Nkx6.1
En1
LHX9
Lhx2
KLF5
Maz
GSC
AMYB
TRPS1
KLF3
AP−2gamma
Klf4
KLF6
AP−2gamma
CUX1
Hnf6b
Cux2
HNF6
AP−2alpha
KLF1
EKLF
PSE
Nkx3.1
Sp2
Nkx2.2
1500
1000
500
0
Median Motif Enrichment −log(pValue)
1
2a
2b
3
4
5
6
Consensus Sequence Block
strand
−
+

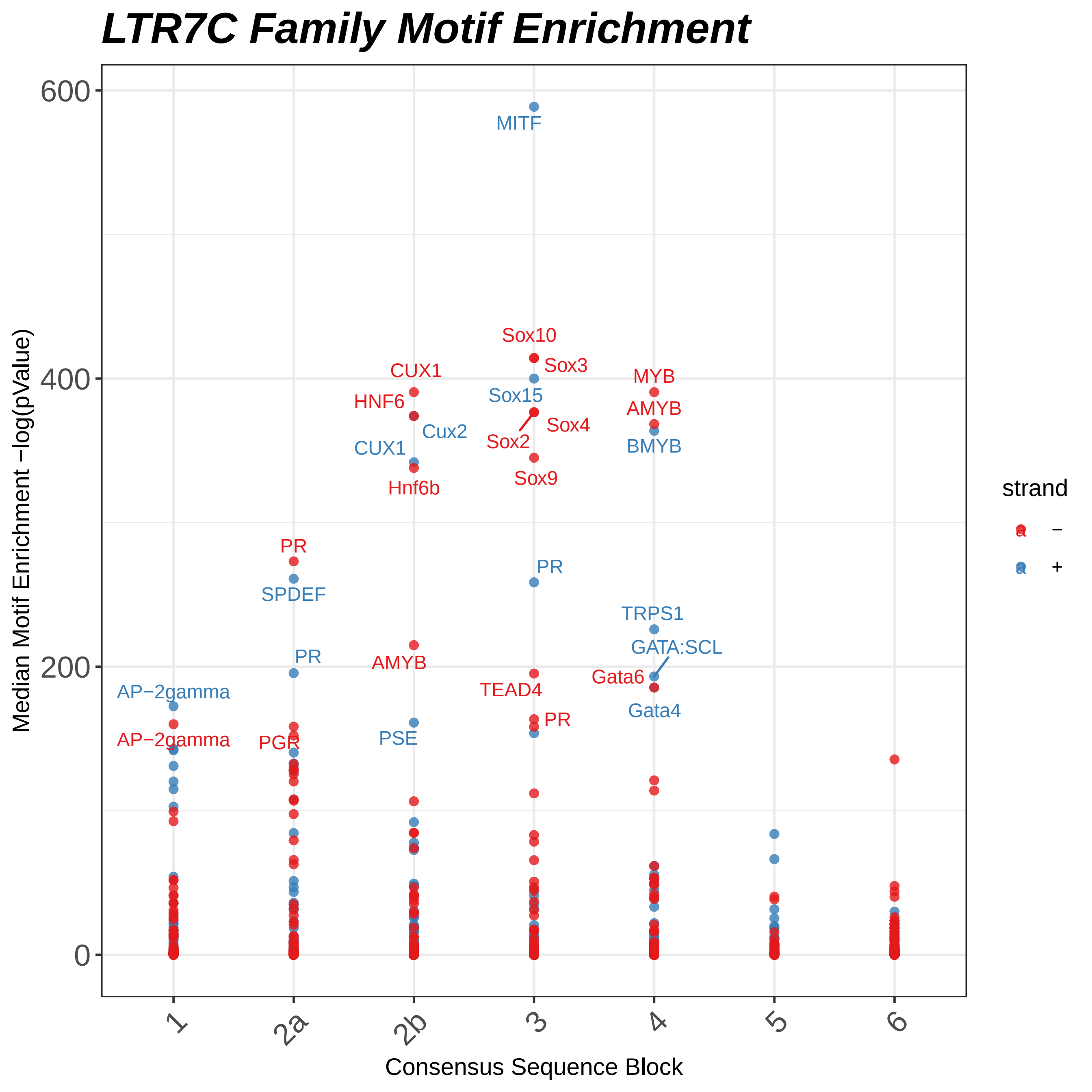
LTR7C Family Motif Enrichment
MITF
Sox10
Sox3
Sox15
Sox4
Sox2
Sox9
CUX1
HNF6
Cux2
CUX1
Hnf6b
MYB
AMYB
BMYB
PR
SPDEF
PR
TRPS1
GATA:SCL
Gata6
Gata4
PR
AMYB
TEAD4
PR
AP−2gamma
AP−2gamma
PGR
PSE
600
400
200
0
Median Motif Enrichment −log(pValue)
1
2a
2b
3
4
5
6
Consensus Sequence Block
strand
−
+

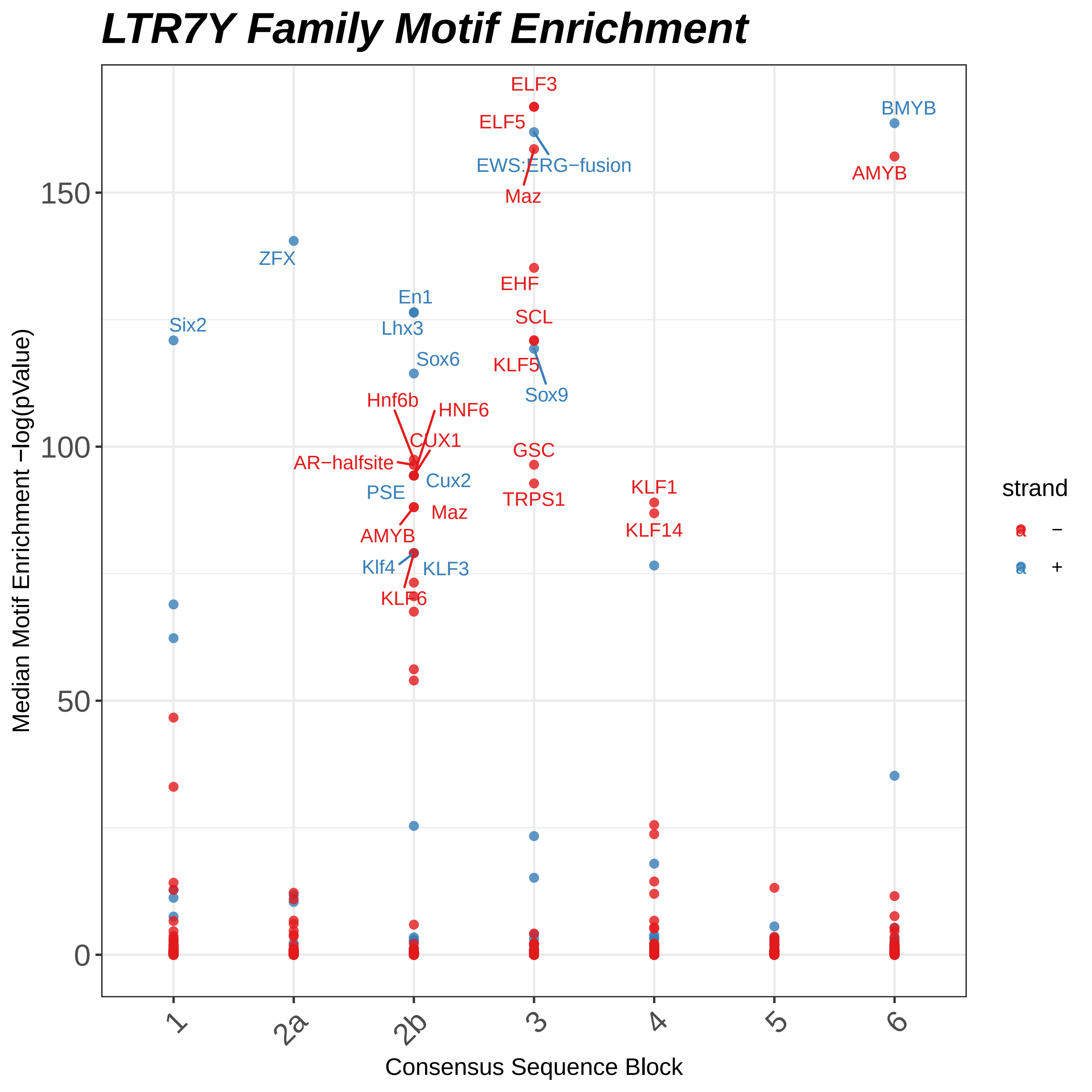
LTR7Y Family Motif Enrichment
Median Motif Enrichment −log(pValue)
Consensus Sequence Block
0
50
100
150
1
2a
2b
3
4
5
6
strand
−
+
ELF3
ELF5
EWS:ERG−fusion
Maz
BMYB
AMYB
ZFX
En1
Lhx3
Six2
EHF
SCL
Sox6
KLF5
Sox9
Hnf6b
HNF6
CUX1
AR−halfsite
Cux2
PSE
GSC
TRPS1
KLF1
KLF14
Maz
AMYB
Klf4
KLF3
KLF6
